# Comparative Study on the Elucidation of Sedimentary Phosphorus Species Using Two Methods, the SMT and SEDEX Methods

**DOI:** 10.1155/2020/8548126

**Published:** 2020-01-22

**Authors:** Pei Sun Loh, Chen-Yu Ying, Hussien Ibrahim Mohammed Alnoor, Xing-Rui Huang, Zhang-Hua Lou, Xue-Gang Chen, Shuangyan He, Zong-Pei Jiang, Ai-Min Jin

**Affiliations:** Ocean College, Zhejiang University, Zhoushan 316021, China

## Abstract

Sedimentary phosphorus (P) forms are important representatives of P sources and their bioavailability as well as the potential of sediments to release P in water. In this study, surface sediments along a transect of the Changjiang Estuary and two transects along the Andong salt marsh in the southwest of Hangzhou Bay were subjected to the elucidation of sedimentary P species using the standards, measurements, and testing (SMT) and sequential extraction (SEDEX) methods. The results showed that the mean sedimentary P forms elucidated by the SMT method were as follows: organic P (OP; ∼11–14 mg/kg; ∼30–45% of total P; TP) > apatite P (∼5–15 mg/kg; ∼21–36% TP) > Fe/Al-P (∼8–14 mg/kg; ∼31–34% TP), with inorganic P (IP) composing 54–70% of TP. The mean sedimentary P forms elucidated by the SEDEX method were as follows: authigenic P (∼54–68 mg/kg; ∼41–46% TP) > extractable P (Ex-P; ∼36–53 mg/kg; ∼28–34%) > Fe-P (∼21–27 mg/kg; ∼13–19%) > OP (∼8.7–13 mg/kg; ∼5–8%) > detrital P (De-P; ∼2 mg/kg; ∼1–2% TP), with IP composed of ∼91–94% TP. These results showed that the SEDEX method elucidated higher concentrations of sedimentary P forms as well as the TP from these coastal sediments although the SMT method had the advantage of being more economic and faster. The results of both the SMT and SEDEX methods showed that the Andong salt marsh and Changjiang Estuary sediments had much bioavailable P. The mean percentages of bioavailable P from the SMT and SEDEX methods were ∼64–74% and 52–56% of TP, respectively, indicating that these sediments were prone to release P to the coastal areas.

## 1. Introduction

Sediment samples are usually subjected to a series of sequential extraction processes to elucidate different phosphorus (P) forms. One of the early sequential extraction processes was developed by Psenner [1] to separate the P in sediments into five main fractions as follows: (i) loosely bound, ion-exchangeable, pore-water P, or NH_4_Cl-P fraction; (ii) P bound to reducible forms of iron or bicarbonate-dithionite (Fe)-P; (iii) P bound to hydrated oxides of Al and nonreducible Fe as well as P in organisms and organic P (OP) or NaOH-P; (iv) calcium-bound, apatite P (Ca-P or authigenic P or HCl-P); (v) refractory and other organically bound P [2–4]. Another sequential extraction process was then developed by Ruttenberg [5], which was eventually known as the sequential extraction (SEDEX) method, to quantify different sedimentary P forms into loosely bound P (Ex-P), Fe-bound P (Fe-P), authigenic P, detrital P, and OP. Thus, the fractions of P elucidated by Psenner [1] and Ruttenberg's [5] methods were similar with the following exceptions: the P bound to hydrated oxides of Al and nonreducible Fe as well as the P in organisms and the OP or NaOH-P fraction elucidated by Psenner's method and the detrital P fraction elucidated by Ruttenberg's method. These methods were followed by the elucidation of NaOH-extractable P (NaOH-P), which is the P fraction bound to Al, Fe, Mn oxides and hydroxides, HCl-P or Ca-bound P, OP, IP, and TP using the standards, measurements, and testing (SMT) method by Ruban et al. [6, 7]. Thus, the NaOH-P in the SMT method is equivalent to the Ex-P and Fe-P forms in the SEDEX method, and the HCl-P in the SMT method is equivalent to the authigenic P fraction extracted by the SEDEX method.

Among these sedimentary P fractions, loosely bound P is loosely sorbed, exchangeable, and water soluble; therefore, it is the most labile fraction. Fe-P is pH- and redox-sensitive. It is a source of internal P loading during anoxic conditions. OP is released as a phosphate during the aerobic decomposition of organic matter. Authigenic P is the most stable P form. Although it is not bioavailable, it could serve as a tracer in determining the sources of organic matter that represent materials from erosion processes. Ex-P, Fe-P, and OP are considered exchangeable or bioavailable [4, 8–11].

Several previous studies used the SMT and SEDEX methods to elucidate sedimentary P species. Examples of studies that have used the SEDEX method are presented here. Meng et al. [12] determined the sedimentary P species in the surface sediments along the Changjiang Estuary and in the East China Sea and found that the highest De-P in these areas indicated the importance of riverine inputs. Adhikari et al. [13] elucidated the sedimentary P species in sediment cores collected from the Gulf of Mexico and found the importance of internal P loading in contributing to the bioavailable P. Higher De-P in the surface sediments in the central Pacific Ocean indicated the contribution of refractory P from the atmospheric input. It was also found that the labile P could have been released into the water because of the decomposition of organic matter during particle settling [14]. Similar studies found that the shellfish aquaculture did not cause significant changes in the sedimentary P species in Sishili Bay [15] but that seasonal variations caused changes in the sedimentary P species in the East China Sea [16]. The Ex-P, authigenic P, De-P, IP, and TP in the surface and core sediments of the eastern Hainan Island in the South China Sea were affected by particle size [17]. High OC/OP ratios indicated that the surface sediments in the Caspian Sea were terrestrial in origin [18]. Some studies used the SMT method to elucidate the sedimentary P species in various environments. Studies of sedimentary P forms in the Bay of Seine and the Loire and Gironde Estuaries [19] and the Gulf of Gdańnsk [20] found that the Fe/Al-P fraction and TP concentrations were mainly due to fine particles. The Loire and Gironde Estuaries, which frequently showed hypoxia, had lower Fe/Al-P fractions than the Bay of Seine did [19]. Similar studies found low pollution in the Quanzhou Bay Estuarine Wetland [21], but high P pollution in rivers around the Bohai Sea [22].

In this study, surface sediments along a transect of the Changjiang Estuary and two transects along the Andong salt marsh in the southwest of Hangzhou Bay were subjected to the SMT and SEDEX methods to elucidate sedimentary P species. The objective of this study was to determine which method was more suitable in elucidating P species in the sediments in these coastal zones.

## 2. Materials and Methods

### 2.1. Sediment Sampling and Pretreatment

Surface sediments were collected manually by scooping into plastic bags approximately 5 cm of the surface sediments in two transects in the Andong salt marsh system, which is situated near the town of Andong in the southwestern part of Hangzhou Bay, China. There were eight sampling locations in Transect A (AD-A) and eight sampling locations in Transect C (AD-C). Surface sediments were collected from the Changjiang Estuary using a grab sampler that collected samples from the surface to a depth of approximately 5 cm to 10 cm. The locations in the Andong salt marsh and Changjiang Estuary were previously reported in Yuan et al. [23]. The sediments were immediately transported to the laboratory. The wet sediments were frozen, stored, and then dried at 45°C for three days.

### 2.2. Analytical Methods

Different sedimentary P forms were extracted using the standards, measurements, and testing (SMT) method by Ruban et al. [6, 7] and following Cheng et al. [24]. A total of 200 mg of dry sediment was weighed into a centrifuge tube, followed by addition of 20 ml of 1 M NaOH. The sediment in NaOH was shook for 16 h, after which the sample was centrifuged at 2000*g* for 15 min and the supernatant was measured for NaOH-P or Fe/Al-P. The sediment residue was then washed with 12 ml of 1 M NaCl, stirred for 5 min and centrifuged at 2000*g* for 15 min. The supernatant from this step was discarded. The residue was added with 1 M HCl and was extracted for 16 h. The solution was then centrifuged at 2,000*g* for 15 min, and the supernatant from this step was measured for HCl-P. A separate 200 mg of the dry sediment were combusted at 450°C for 3 h. When it was cool, the ash was poured into a centrifuge tube and added with 1 M HCl. The solution was extracted for 16 h, centrifuged at 2000*g* for 15 min, and the supernatant was determined for OP. The concentrations of all P species were determined as the molybdenum blue complex using the UV-visible spectrophotometer UV-8000 (METASH, Shanghai, China) by measuring the absorbance at a wavelength of 885 nm. IP is the sum of NaOH-P and HCl-P, and TP is the sum of OP and IP.

Using Ruttenberg's [5] method, in the first P fraction, 0.5 g of dry sediment was weighed into a 50 ml centrifuge tube, 20 ml of MgCl_2_ was added, and the solution was adjusted to pH 8 with Na_4_OH. This was extracted by shaking for 2 h at room temperature, after which the content was centrifuged and the supernatant was saved. The residue and another 20 ml MgCl_2_ were added and shaken for 2 h, centrifuged, and the supernatant was saved. The supernatant derived in this step will be determined for the Ex-P fraction. In the second part, the residue from the first part was added to 20 ml of citrate-dithionite-bicarbonate (CDB) solution. The extraction was carried out by shaking for 8 h at room temperature. The content was then centrifuged, and the supernatant was saved. The residue was added to 20 ml MgCl_2_ and shaken for 2 h, centrifuged, and the supernatant was saved. The residue was then washed with 10 ml H_2_O for 2 h, centrifuged, and the supernatant saved. The supernatant from this part was measured for Fe-P. The residue was then added to 20 ml pH 4 acetate buffer and shaken for 6 h at room temperature, after which the mixture was centrifuged and the supernatant was saved. The residue was then washed twice with MgCl_2_, centrifuged, and the supernatant was saved. Finally, the residue was washed with 10 ml H_2_O and centrifuged; the supernatant was saved. The supernatant from this step will be measured for authigenic P. In the next step, the residue was added to 1 M HCl and shaken for 16 h, after which the content was centrifuged and the supernatant was saved to be analysed for the presence of De-P. After this process, the residue was moved to a crucible and dried in an oven at 80°C for one day. It was then combusted at 550°C for 5 h. The residue was cooled, added to 1 M HCl, and shaken for 16 h. The supernatant from this step will be measured for OP. Inorganic P (IP) was the sum of Ex-P + Fe-P + authigenic P + De-P. Total P (TP) was the sum of IP and OP. All P concentrations were determined colourimetrically to be a molybdenum blue complex. The absorbance was measured at a wavelength of 885 nm using a UV-visible spectrophotometer UV-8000 (METASH, Shanghai, China).

## 3. Results and Discussion

The results of the sedimentary P forms elucidated using the SMT and SEDEX methods are shown in Tables 1 and 2. The mean sedimentary TP concentrations and the mean sedimentary P fractions in the study location elucidated using the SMT method are as follows:

  TP: AD-C (41.73 mg/kg) > CE (39.33 mg/kg) > AD-A (24.89 mg/kg)
  CE: OP (13.84 mg/kg; 33.91%) > HCl-P (13.33 mg/kg; 35.08%) > Fe/Al-P (12.16 mg/kg; 31.01%)
  AD-A: OP (11.07 mg/kg; 45.28%) > Fe/Al-P (8.56 mg/kg; 33.20%) > HCl-P (5.06 mg/kg; 21.52%)
  AD-C: HCl-P (14.89 mg/kg; 35.59%) > Fe/Al-P (14.20 mg/kg; 34.19%) > OP (12.64 mg/kg; 30.22%)

The mean sedimentary TP concentrations and the mean sedimentary P fractions in the study location elucidated using the SEDEX method are as follows:

  TP: AD-C (159.86 mg/kg) > CE (151.13 mg/kg) > AD-A (127.33 mg/kg)
  CE: authigenic P (68.39 mg/kg; 45.67%) > Ex-P (46.59 mg/kg; 30.64%) > Fe-P (21.04 mg/kg; 13.78%) > OP (12.53 mg/kg; 8.16%) > De-P (2.58 mg/kg; 1.74%)
  AD-A: authigenic P (54.93 mg/kg; 42.83%) > Ex-P (36.21 mg/kg; 28.93%) > Fe-P (24.86 mg/kg; 19.23%) > OP (8.70 mg/kg; 6.86%) > De-P (2.63 mg/kg; 2.10%)
  AD-C: authigenic P (67.00 mg/kg; 41.77%) > Ex-P (53.45 mg/kg; 33.77%) > Fe-P (27.35 mg/kg; 16.85%) > OP (9.24 mg/kg; 5.83%) > De-P (2.83 mg/kg; 1.79%)

These results showed that the TP concentrations elucidated by the SEDEX method were three to five times higher than those elucidated by the SMT method at 118.13 mg/kg, 111.80 mg/kg, and 102.44 mg/kg in AD-C, CE, and AD-A, respectively. The OP concentrations extracted by the SMT method (ranging from 4.97 mg/kg to 25.80 mg/kg; Table 1) were slightly higher than the OP extracted by the SEDEX method (ranging from 3.05 mg/kg to 18.11 mg/kg; Table 2). The reason is that in the SMT method, the determination of OP was conducted on a new batch of sediment samples. In the SEDEX method, the determination of OP was carried out using the residue from the previous steps; hence, these differences were negligible. The differences of mean OP concentrations between both methods for AD-A, AD-C, and CE are 2.57 mg/kg (22.8%), 3.4 mg/kg (26.9%), and 1.31 mg/kg (9.5%), i.e., the SMT method produced a mean of 20% higher OP than the SEDEX method. In the SEDEX method, the OP fraction is determined during the fifth step of the sequential extraction process. In the SMT method, a new batch of sediment is subjected to determination of the IP and OP fractions. Considering that in the SEDEX method, OP is determined from the residue after four previous extraction steps, whereas the OP in the SMT method is determined from a new batch of sample, we would say that the OP concentration elucidated by the SEDEX method could be representative of the OP in the sediment.

**Figure 1:**
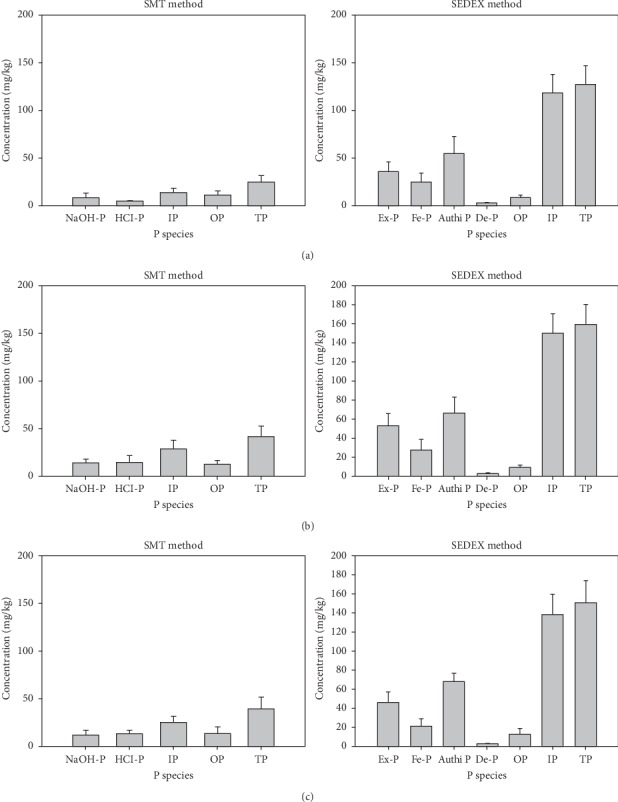
Comparison of sedimentary P species elucidated by the SMT and SEDEX methods for (a) AD-A, (b) AD-B, and (c) CE.

The authigenic P elucidated by the SMT method was the second highest portion of the TP (ranging from 8 mg/kg to 14 mg/kg, representing ∼33% to 35% of TP), whereas the authigenic P elucidated by the SEDEX method was the highest portion of TP (ranging from 54 mg/kg to 68 mg/kg, representing ∼41% to 46% of TP). Although the composition percentages of authigenic P to TP determined by both methods were similar, the concentrations of authigenic P extracted by the SEDEX method were around four to seven times higher than those extracted by the SMT method. The Fe/Al-P fraction extracted by the SMT method was equivalent to the Ex-P and Fe-P fractions extracted by the SEDEX method. The Fe/Al-P concentrations in CE, AD-A, and AD-C were 12.16 mg/kg, 8.56 mg/kg, and 14.20 mg/kg, representing 31.01%, 33.20%, and 34.19%, respectively. The Ex-P concentrations were 46.59 mg/kg, 36.21 mg/kg, and 53.45 mg/kg, representing 30.64%, 28.93%, and 33.77%, respectively. The Fe-P concentrations were 21.04 mg/kg, 24.86 mg/kg, and 27.35 mg/kg, representing 13.78%, 19.23%, and 16.85% in CE, AD-A, and AD-C, respectively. The combinations of Ex-P and Fe-P in CE, AD-A, and AD-C were 67.63 mg/kg, 61.07 mg/kg, and 80.8 mg/kg, representing 44.42%, 48.16%, and 50.62%, respectively. The combination of Ex-P and Fe-P was five to seven times higher than the Fe/Al-P fraction. These results showed that TP and all P fractions, except the OP, were higher when they were elucidated by the SEDEX compared to the SMT method (Figure 1). The SMT method has been utilised to elucidate different P species in freshwater sediments, and the results have been reproducible [6, 7]. Moreover, this method is economical and simple to use. Our results showing that lower concentrations of P forms were extracted by the SMT method indicate that this method may be more suitable in freshwater sediments.

In our study areas, the surface sedimentary TP was lower compared to the TP in other locations that were elucidated using the SEDEX method. The results showed the following: TP from 416.5 to 638.5 mg/kg in the East China Sea sediment cores [25]; TP from 465 to 663.4 mg/kg in the Changjiang Estuary and adjacent East China Sea surface sediments [12]; TP from 474 to 1,035 mg/kg in the northern Gulf of Mexico sediment cores [13]; TP from 409.2 to 3,689 mg/kg in the central Pacific Ocean surface sediments [14]; TP of 466.24–669.29 mg/kg in the Sishili Bay, China [15], TP from 246.76 to 692.54 mg/kg at the eastern coast of Hainan Island surface sediment [17]; TP from 369.83 to 739.97 mg/kg in the East China Sea core sediments [26]; TP from 431 to 594 mg/kg in the Caspian Sea surface sediments [18]. Similarly, in our study areas, the surface sedimentary TPs were lower compared to the sedimentary TPs in other locations elucidated using the SMT method: TP ranging from 520.8 to 1376.4 mg/kg in the Loire Estuary; from 130.2 to 787.4 mg/kg in the Gironde Estuary; from 189.1 to 576.6 mg/kg in the Bay of Seine [19]; at 412.61 mg/kg in the Gulf of Gdańnsk [20]; from 217.8 to 1,640 mg/kg in the lakes in the mid and lower Changjiang River [27]; from 303.87 to 761.59 mg/kg in the subtropical wetland reserve in southeast China [21]; 87.28–2610.43 mg/kg in the main rivers discharging into the Bohai Sea [22]. Thus, the sedimentary TP found in our study areas indicates that these locations were less polluted compared to most other areas.

The bioavailable P (OP + Fe/Al-P) elucidated by the SMT method ranged from 9.8 to 43.9 mg/kg, representing 46.17–85.15% of TP. OP was composed of 20.08–68.34% TP. OP could have been released into water during the decomposition of organic matter. Fe/Al-P (or NaOH-P) comprised 6.77–52.78% of TP. This fraction was comprised of the P adsorbed to iron or aluminium. Iron-bound P could be released into water under anoxic conditions although we do not know the amount of iron-bound P in this Fe/Al-P fraction. The bioavailable P (Ex-P + Fe-P + OP) extracted by the SEDEX method ranged from 46.30 to 106.11 mg/kg, representing 43.09–66.50% of TP. Of this, Ex-P was composed of 14.85–43.19% TP, Fe-P was composed of 6.31%–31.50% of TP, and OP was composed of 2.51–12.86% of TP, indicating that these sediments could be released from the Ex-P and OP fractions by the decomposition of organic matter and from the Fe-P during anoxic conditions. In these study areas, the bioavailable P fraction elucidated by both methods was higher than in other locations, such as the East China Sea, which had 6–32% bioavailable P to TP [12, 23]; Sishili Bay, China, which had 26.7–35.5% bioavailable P [15]; the eastern coast of Hainan Island, China, which had 20.3–54.2% bioavailable P [17]; the Caspian Sea, which had a mean of 37.2% bioavailable P [18]. Thus, based on the results of the present study, the Andong salt marsh and the Changjiang Estuary released P during the decomposition of organic matter and under anoxic conditions, which could be utilised by microorganisms.

If we substitute the results of OP concentrations from the SMT method as the SEDEX OP concentrations, the sequence of mean sedimentary P forms elucidated by the SEDEX method is still the same, as follows: authigenic P > Ex-P > Fe-P > OP > De-P. Of the P forms elucidated from the SMT method, OP was the highest fraction in CE and AD-A, followed by the HCl-P and Fe/Al-P in CE, but among Fe/Al-P and HCl-P in AD-A; HCl-P was the highest P fraction, followed by FE/Al-P and OP in AD-C. These results indicate that overall the SMT method has underestimated the inorganic P forms. Besides, the P forms extracted by the SMT method also showed higher variability in terms of the sequence of their abundances.

## 4. Conclusion

In this study, surface sediments along the Changjiang Estuary and the Andong salt marsh in the southwest of Hangzhou Bay were subjected to the elucidation of sedimentary P species using the SMT and SEDEX methods. The ranges of sedimentary P species elucidated by the SMT method were as follows: OP (5.00–25.80 mg/kg) > HCl-P (4.10–28.94 mg/kg) > NaOH-P (1.35–20.05 mg/kg). The overall IP and TP ranged from 6.31 to 42.64 mg/kg and from 13.90 to 60.30 mg/kg, respectively. The ranges of sedimentary P species elucidated by the SEDEX method were as follows: authigenic P (37.98–91.54 mg/kg) > Ex-P (20.68–71.43 mg/kg) > Fe-P (9.2–50.6 mg/kg) > OP (3.05–18.55 mg/kg) > De-P (1.95–3.78 mg/kg). IP and TP ranged from 86.67 to 188.41 mg/kg and from 91.30 to 196.99 mg/kg, respectively. The SEDEX method elucidated higher concentrations of all P forms (except OP), which indicates that it is the most suitable method for elucidating sedimentary P species in samples of these coastal sediments. Another reason for choosing the SEDEX method is that more P forms are elucidated, as follows: Ex-P (exchangeable P or loosely bound P), Fe-P, authigenic P, detrital P, and OP (IP = Ex-P + Fe-P + authigenic P + De-P), in comparison to the SMT method which elucidated the NaOH-P, Ca-P, IP, and OP. A third reason for choosing the SEDEX method is that this method separates the inorganic phosphorus more completely into four fractions, whereas the SMT method has the NaOH-P fraction which may contain the Ex-P and Fe-P, and this method does not elucidate the detrital fraction. This finding is consistent with previous studies that determined the suitability of the SMT method for freshwater sediments. However, in the present study, sediment samples from only two coastal areas were analysed. Further studies are needed, especially comparisons between sediments in distinctively different environments, such as freshwater and seawater.

## Figures and Tables

**Table 1:** Sedimentary P species as elucidated from the SMT method.

	Concentration (mg/kg)						Percentage (%)				
	NaOH-P	HCl-P	IP	OP	TP	BAP	NaOH	HCl-P	IP	OP	BAP
*Location-AD*											
A8	8.50	5.90	14.40	19.50	33.90	28.00	25.07	17.40	42.48	57.52	82.60
A7	1.35	4.96	6.31	13.62	19.93	14.97	6.77	24.89	31.66	68.34	75.11
A6	7.73	4.60	12.33	13.65	25.98	21.38	29.75	17.71	47.46	52.54	82.29
A5	9.27	5.70	14.97	11.30	26.27	20.57	35.29	21.70	56.99	43.01	78.30
A4	17.35	4.88	22.23	10.64	32.87	27.99	52.78	14.85	67.63	32.27	85.15
A3	13.63	4.48	18.11	7.88	25.99	21.51	52.44	17.24	69.68	30.32	82.76
A2	4.80	4.10	8.90	5.00	13.90	9.80	34.53	29.50	64.03	35.97	70.50
A1	5.86	5.86	11.72	8.54	20.26	14.40	28.92	28.92	57.85	42.15	71.08
Mean	8.56	5.06	13.62	11.27	24.89	19.83	33.20	21.52	54.72	45.28	74.48
Stdev	5.04	0.68	5.04	4.43	6.72	6.47	14.90	5.66	13.24	13.24	5.66
C10	13.50	17.30	30.80	10.30	41.10	23.80	32.85	42.09	74.94	25.06	57.91
C9	8.13	13.08	21.21	10.25	31.46	18.38	25.84	41.58	67.42	32.58	58.42
C8	13.70	28.94	42.64	11.12	53.76	24.82	25.48	53.83	79.32	20.68	46.17
C7	18.50	16.90	35.40	15.90	51.30	34.40	36.06	32.94	69.01	30.99	67.06
C6	20.05	16.83	36.88	18.59	55.47	38.64	36.15	30.34	66.49	33.51	69.66
C3	9.52	10.26	19.78	4.97	24.75	14.49	38.46	41.45	79.92	20.08	58.55
C2	12.10	9.58	21.68	13.60	35.28	25.70	34.30	27.15	61.45	38.55	72.85
C1	18.09	6.23	24.32	16.42	40.74	34.51	44.40	15.29	59.70	40.30	84.71
Mean	14.20	14.89	29.09	12.64	41.73	26.84	34.19	35.59	69.78	30.22	64.41
Stdev	4.34	6.96	8.56	4.37	11.10	8.40	6.29	11.73	7.64	7.64	11.73

*Location-CE*											
20	9.66	9.01	18.67	6.34	25.01	16.00	38.62	36.03	74.65	25.35	63.97
4	10.60	9.80	20.40	15.10	35.50	25.70	29.86	27.61	57.46	42.54	72.39
6	6.77	14.41	21.18	8.76	29.94	15.53	22.61	48.13	70.74	29.26	51.87
11	17.32	13.17	30.49	13.36	43.85	30.68	39.50	30.03	69.53	30.47	69.97
13	18.10	16.40	34.50	25.80	60.30	43.90	30.02	27.20	57.21	42.79	72.80
21	10.53	17.16	27.69	13.69	41.38	24.22	25.45	41.47	66.92	33.08	58.53
Mean	12.16	13.33	25.49	13.84	39.33	26.01	31.01	35.08	66.09	33.91	64.92
Stdev	4.52	3.36	6.36	6.74	12.43	10.54	6.84	8.43	7.22	7.22	8.43

**Table 2:** Sedimentary P species as elucidated from the SEDEX method.

	Concentration (mg/kg)								Percentage (%)						
	Ex-P	Fe-P	Authi P	De-P	OP	IP	TP	BAP	Ex-P	Fe-P	Authi P	De-P	OP	IP	BAP
*Location-AD*															
AD	49.49	20.14	37.98	3.13	11.98	110.74	122.72	81.61	40.33	16.41	30.95	2.55	9.76	90.24	66.50
A7	42.87	32.00	70.57	3.56	10.47	149.00	159.47	85.34	26.88	20.07	44.25	2.23	6.57	93.43	53.51
A6	44.48	18.21	39.93	2.84	11.13	105.46	116.59	73.82	38.15	15.62	34.25	2.44	9.55	90.45	63.32
A5	42.41	19.09	52.75	2.08	9.32	116.33	125.65	70.82	33.75	15.19	41.98	1.66	7.42	92.58	56.36
A4	31.30	34.27	58.94	2.30	9.40	126.81	136.21	74.97	22.98	25.16	43.27	1.69	6.90	93.10	55.04
A3	29.21	12.46	42.53	2.47	4.63	86.67	91.30	46.30	31.99	13.65	46.58	2.71	5.07	94.93	50.71
A2	20.68	22.60	89.58	1.98	4.45	134.84	139.29	47.73	14.85	16.23	64.31	1.42	3.19	96.81	34.27
A1	29.22	40.13	47.15	2.67	8.21	119.17	127.38	77.56	22.94	31.50	37.02	2.10	6.45	93.55	60.89
Mean	36.21	24.86	54.93	2.63	8.70	118.63	127.33	69.77	28.98	19.23	42.83	2.10	6.86	93.14	55.08
Stdev	9.93	9.50	17.70	0.54	2.82	18.95	19.61	14.76	8.63	6.14	1.19	0.47	2.17	2.17	9.92
C10	42.63	21.53	79.53	2.09	8.85	145.78	154.63	73.01	27.57	13.92	51.43	1.35	5.72	94.28	47.22
C9	66.56	25.81	51.99	1.95	7.79	146.31	154.10	100.16	43.19	16.75	33.74	1.27	5.06	94.94	65.00
C8	62.38	33.24	52.90	2.20	9.00	150.72	159.72	104.62	39.06	20.81	33.12	1.38	5.63	94.37	65.50
C7	71.43	16.78	74.02	2.91	13.47	165.14	178.61	101.68	39.99	9.39	41.44	1.63	7.54	92.46	56.93
C6	56.67	22.94	45.23	3.54	11.45	128.38	139.83	91.06	40.53	16.41	32.35	2.53	8.19	91.81	65.12
C3	41.38	16.72	64.19	2.77	7.44	125.06	132.50	65.54	31.23	12.62	48.45	2.09	5.62	94.38	49.46
C2	42.84	50.6	91.54	3.43	8.58	188.41	196.99	102.02	21.75	25.69	46.47	1.74	4.36	95.64	51.79
C1	43.55	31.17	76.60	3.78	7.36	155.10	162.46	82.08	26.81	19.19	47.15	2.33	4.53	95.47	50.52
Mean	53.45	27.35	67.00	2.83	9.24	150.61	159.86	90.02	33.77	16.85	41.77	1.79	5.83	94.17	56.44
Stdev	12.30	11.15	16.07	0.71	2.15	20.17	20.52	14.87	7.92	5.09	7.72	0.48	1.37	1.37	7.76

*Location-CE*															
20	33.38	15.94	66.75	2.43	3.05	118.50	121.55	52.37	27.46	13.11	54.92	2.00	2.51	97.49	43.09
4	46.72	9.20	73.02	2.80	13.98	131.74	145.72	69.90	32.06	6.31	50.11	1.92	9.59	90.41	47.97
6	47.91	26.90	66.45	2.69	6.71	143.95	150.66	81.52	31.80	17.85	44.11	1.79	4.45	95.55	54.11
11	60.13	31.19	83.05	2.45	14.79	176.82	191.61	106.11	31.38	16.28	43.34	1.28	7.72	92.28	55.38
13	55.36	17.74	59.61	2.14	18.11	134.85	152.96	91.21	36.19	11.60	38.97	1.40	11.84	88.16	59.63
21	36.03	25.28	61.45	2.97	18.55	125.73	144.28	79.86	24.97	17.52	42.59	2.06	12.86	87.14	55.35
Mean	46.59	21.04	68.39	2.58	12.53	138.60	151.13	80.16	30.64	13.78	45.67	1.74	8.16	91.84	52.59
Stdev	10.47	8.15	8.58	0.30	6.30	20.59	22.77	18.30	3.92	4.42	5.79	0.33	4.09	4.09	5.98

## Data Availability

All data used to support the findings of this study are presented in this article.
